# Weaving words for textile museums: the development of the linked SILKNOW thesaurus

**DOI:** 10.1186/s40494-022-00681-x

**Published:** 2022-05-10

**Authors:** Ester Alba, Mar Gaitán, Arabella León, Dunia Mladenić, Janez Brank

**Affiliations:** 1 Department of Art History, Universitat de València, Av. de Blasco Ibáñez, 28, 46010 Valencia, Valencia Spain; 2 Artificial Intelligence Laboratory, Jozef Stefan Institute, Jamova cesta 39, 1000 Ljubljana, Slovenia

**Keywords:** Thesaurus system, Silk heritage, Knowledge platform, Data curation

## Abstract

The cultural heritage domain in general and silk textiles, in particular, are characterized by large, rich and heterogeneous data sets. Silk heritage vocabulary comes from multiple sources that have been mixed up across time and space. This has led to the use of different terminology in specialized organizations in order to describe their artefacts. This makes data interoperability between independent catalogues very difficult. To address these issues, SILKNOW created a multilingual thesaurus related to silk textiles. It was carried out by experts in textile terminology and art historians and computationally implemented by experts in text mining, multi-/cross-linguality and semantic extraction from text. This paper presents the rationale behind the realization of this thesaurus.

## Introduction

Cultural heritage domain is formed by the sum of several tangible and intangible elements that GLAMs strive to preserve, conserve and disseminate. This great variety of cultural property forms, in turn, a large, rich and heterogeneous datasets where different organizations use different terminology to describe their objects [1, 2]. In this sense, a museum can be understood as a large database where cultural heritage objects are guarded for their conservation and dissemination. In order to do so, this database must be able to correctly name each record, especially when this named object enters an inventory and subsequently into a catalogue which has to be sufficiently recognized. Therefore, naming properly a cultural asset also means giving it the capacity to be shared, studied and compared with other similar objects.

It is well known that these institutions strive with a vast amount of cultural heritage that will be named and classified accordingly to their collections, this means that if a collection is based on fine arts objects it will stand their aesthetical and historical aspects whereas if it is an ethnographic collection, they will stand out their anthropological aspects. Therefore, it is safe to say that each museum has generated its own system of classification and inventory of its collections. This is a direct legacy that depends on schools, nations and curators who have carried out this cataloguing from a scholarly point of view [3]. That is why having proper terminology stands out as one fundamental pillar [4]. Control tools such as thesauri, folksonomies and taxonomies, among others, emerge as tools for information retrieval as well as data interoperability. Indeed, controlled vocabularies are essential to provide access to museum collections not only to inside users (curatorial departments, conservators, education department) but also to external users who wish to know more about a subject without knowing the specific term of its search [5]. Moreover, controlled vocabularies are useful to share cultural heritage objects among other institutions, especially in a post-pandemic world, where digitization and open access have demonstrated to be the essentials to provide culture to worldwide audiences.

In this regard, a thesaurus is defined as a controlled vocabulary that has a semantic network of unique concepts [6] that enhances to retrieve information as it is based on queries based on categorized deductions [7]. Thesauri also can link an object with a user, that is, through the use of natural language that facilitates them to conduct any research about cultural assets. Moreover, the vast amount of data associated permits not only to document and describe the object, but it also allows to find likeness or differences between similar cultural assets and associate them, facilitating users to find new connections among them [8]. In addition, connecting a thesaurus to other databases such as other thesauri or wikidata, makes it much more effective, allowing for the exploration of parallel relationships, helping researchers and users in general to develop new research. Moreover, their interoperability is essential nowadays, especially when open databases are essential between cultural institutions, as the current COVID-19 pandemic has demonstrated.

As per heritage related thesauri, we can find some open-access thesauri that provide solutions to specific problems such as ICONCLASS [9] a system designed for art and iconography, available in four languages (English, German, French and Italian) and available as Linked Open Data that ordains hierarchically subjects represented in images. There are others more ambitious when conserving and curating broader collections. In North America, the Canadian Heritage Information Network uses the Humanities Data Dictionary as their metadata standard that allows Canadian museums not only to normalize their vocabulary but also share their collections, in this sense, these metadata standards were based in CIDOC Information Categories. In the same Continent, we can find one of the most standing examples are the Getty vocabularies which are: the Cultural Objects Naming Authorities Thesaurus (CONA), the Iconography Thesaurus (IA), the Geographical Names Thesaurus (TGN), the list of artists’ names in the union (ULAN), and the thesaurus of art and architecture (AAT) where terms related to types of work, materials, styles, techniques, etc. are inscribed, this last one consists of approximately 24,500 descriptors, 2750 guide terms and about 20,000 synonyms, or about 47,000 terms if the approximately 16,000 alternative terms are counted [10] and the information can be retrieved in Dutch, German, Spanish and Chinese. Finally, all the Getty vocabularies are freely available and linked in JSON, RDF, N3/Turtle and N-Triples formats for interoperability, making it one of the most widely used thesauri.

In addition to these international thesauri, at European level, we find that national thesauri or vocabularies such as the ones from the Istituto Centrale per il Catalogo e la Documentazione that has two types of vocabularies, ones that act as predefined closed list while the second ones allow cataloguers to add new terms. In France, the Joconde, offers a thesaurus available online, among the subjects offered the user can find list of authors, periods and styles, materials, representation sources, etc. In Spain, the DOMUS system provides terminology control tools that can be classified into two groups: specialized dictionaries used for specific terminology from their corresponding thematic area (ceramics, numismatics and furniture) and generic thesauri, which are applicable to the cataloguing of all types of cultural assets, these thesauri refer to material, techniques, geography, historical toponymy of the Iberian Peninsula, the Balearic and Canary Islands, cultural contexts, Euro-Mediterranean and Near Eastern cultures, and designations of cultural assets. Also, in the Spanish context, the Andalusian Historical Heritage Thesaurus (TPHA) was designed to meet the needs of the databases of the Andalusian Historical Heritage Information System [11].

Regarding textile vocabularies, they are often based on their own collections [12], such as The Textile Museum Thesaurus from the Textile Museum Thesaurus in Washington, D.C. [13] which was created to improve the cataloguing system of the collection, as well as a tool for facilitating the recovery of objects as they deal with almost 17,000 textiles that range from 3000 B.C. to date. While very specific, it is focused on a specific collection from a specific region, that is why their preferred terms are based on North American literature, and their facets are focused on their collection, that is why it allows distinguishing terms related to the structure of the textiles on one side to physical relations of the elements of a textile on the other and the techniques that were used to produce those fabrics. The Europeana Fashion Vocabulary [14] is focused not only on fabrics but also in objects surrounding them such as books or even blogs and websites. While conceived as an AAT extension, it was necessary to create specific subcategories for the fashion world such as clothing, accessories, contextual objects, communication, events, materials, techniques, decoration and agents. This thesaurus is available online in English, French, German, Greek, Italian, Portuguese, Serbian, Spanish and Swedish and in JSON-LD, RDF, SKOS, XML and PDF formats. Another vocabulary of textile-related terms is the ICOM Vocabulary of Basic Terms for Cataloguing Costume, available in English, German, French and Spanish. Created in 2011, this is a vocabulary of terms specific to clothing and related accessories that has a numerical identifier and acts as a mere list of terms. Finally, there are other monolingual vocabularies related to textile heritage, although they are more focused on fashion. Among them is the Lemmario per la Catalogazione dell'Abito e Degli Elementi Vestimentari, which includes typologies from the eighteenth, nineteenth, and twentieth centuries, and also includes structural and decorative components and techniques. Finally, it is worth mentioning the German vocabulary Aufstellungssystematik Kulturanthropologie des Textilen created by the University of Dortmund, which serves as a classification system for the Cultural Anthropology of Textiles of the Emil Figge Library.

Despite silk’s importance worldwide and especially within Europe, to the best of our knowledge, there are not any silk heritage specialized thesauri. There are some specific vocabularies that either are related to fabrics as a whole, or they are focused on textile engineering. To meet these challenges, SILKNOW built a multilingual thesaurus dedicated to the specific vocabulary of historic silk textiles, which will also include local term variants. The thesaurus will help heritage institutions to provide access to and preserve silk heritage in the digital environment. Participating and collaborating institutions will radically improve their cataloguing practices and digital data retention. In addition, the thesaurus will serve as an example of the benefits of shared cataloguing frameworks and data interoperability.

In this paper we extend the research conducted in [15], where we present the initial development of the thesaurus. Whereas in this paper we show the results obtained after 3 years of research and development. The article presents (1) the methodology, content and structure of the thesaurus, including the challenges of a multilingual thesaurus; (2) we introduce for the first time its relationship with other ontologies such as wikidata; (3) the evaluation of the thesaurus, including the use and relation of the concepts included in the thesaurus compared to their use in museums; (4) Conclusions.

## SILKNOW thesaurus

The SILKNOW thesaurus aims to improve silk heritage knowledge by standardizing silk terminology characterized by large, rich and heterogeneous data sets. The objectives are twofold: to create a controlled silk heritage vocabulary that will lead to heritage conservation, and the second one is to support the rest of SILKNOW tools as it is the knowledge basis such as the Virtual Loom [16] which connects historical weaving techniques with their definitions and restrictions or ADASilk, the SILKNOW search engine that embeds a Spatio-temporal map. The SILKNOW thesaurus is targeted to researchers, students and cultural heritage professionals. For example, a researcher may use the thesaurus to connect terms that she or he could have found in historical documentation or whenever a term is identified in a vernacular language. Art history and conservation students will be familiarized with a variety of textile terms. It will also help to disseminate scientifically accurate terms that come from a standardized vocabulary whose final purpose is to improve the understanding, conservation and dissemination of silk heritage.

Silk heritage terminology presents some specific challenges, as the vocabulary that surrounds it comes from various sources that have changed across time and space. Moreover, it changes according to users (weavers or historians), nationalities (Europe or North America), or disciplines (ethnographic specialists vs art historians), etc. [14]. The multilingual SILKNOW thesaurus [15] is a controlled vocabulary that has a semantic network of unique concepts [6], where each of its 666 preferred terms (PT) are controlled, this means that each PT represents a single concept. This thesaurus is unique in its kind as it is the only one entirely dedicated to silk heritage, from the fifteenth to nineteenth centuries, which corresponds to the period that SILKNOW is covering. It includes weaving techniques, materials, depictions and equipment among other things. Innovation comes from the fact that it addresses to the silk heritage area instead of limiting to a specific data source, that is, even though there are few thesaurus or vocabularies related to textiles and silk, there are either too specific (created for a certain collection from a certain museum), or monolingual, or they are not online, or they are not open access. SILKNOW addresses this issue by creating a specific thesaurus, taking into account several museum’s collections, the broad historical and current literature on the subject as well as the importance of industrial terminology.

### Content and structure

The SILKNOW thesaurus followed a mixed method [6], where 80% was inductive (terms were included in the thesaurus as soon as they were found in the literature) and 20% deductive (due to museum records and previous knowledge from the researchers). As mentioned, one of the main objectives was to create a unique thesaurus that would be able to cover the whole silk heritage, which means, to include as many terms as possible that comprises fibres that were used to produce a fabric to motifs and their use. In this sense, it was necessary to include terms that will most likely appear in museums’ catalogues, taking into account that not all museums catalogue the same way, and that the thesaurus could be used by curators, but also by conservators.

On the other hand, materials and techniques were the first terms to be included in the thesaurus as they were needed to develop other SILKNOW tools such as the Virtual Loom [17], to map collections that are part of the Knowledge Graph and to evaluate those tools. Later on, the rest of the terms related to colours, patterns, machinery, clothing, etc. were included. Scope notes and terms followed ISO 25964 regulation of thesaurus. These guidelines established that PT should generally be treated as nouns, except processes and techniques that can be found in the gerund or in the infinitive form. Also, whenever it exists, the etymology of the word should be specified. Centuries and/or periods should be provided whenever they are clear and referenced in the literature. Finally, scope notes should be written in present tense, except when literature specifies a past use for that term, e.g., “during the fifteenth century, this term was used for…”. If a term was polysemic, the rule was to add as many times as it has meanings. In some cases, polysemy was not easy to undertake, as some concepts may refer to a weaving technique and fabric at the same time such as satin or twill, this was solved by adding qualifiers to those terms. In this sense, a list of qualifiers was created to homogenize the thesaurus and make it easier for the user to find the correct term. In some languages, polysemy was especially complicated in English as it is a Germanic language while Spanish is a Romance language. Table 1 shows the use of qualifiers.

**Table 1:** Use of qualifiers in the four languages

Spanish	English	French	Italian
Raso → ligamento	Satin → weave	Satin → armure	Raso → intreccio
Raso → tejido	Satin → fabric	Satin → tissu	Raso → tessuto

The SILKNOW thesaurus contemplates the three basic relations among terms: equivalence, association and hierarchical relations. As for the equivalence relationships that is, whenever different names refer to the same concept as synonyms or quasi-synonyms as shown in Table 2.

**Table 2:** Use of synonyms

Spanish	English	French	Italian
seda cruda → seda grega, seda grege	warp beam → cane roller	quit → soie quite	saia → spinato; spigato

Associative relationships occurred when different terms are closely related conceptually but not hierarchically, these were added especially for machines and the objects that form them.

Beater → Jacquard Loom

Finally, as per hierarchical relationships, the SILKNOW thesaurus uses the AAT as the basic structure for its facets, nevertheless, as the AAT is a generic thesaurus it was not specific enough to place silk terms, we proposed new guide terms:

- Conditions and effects for textiles by their visual aspects: It refers to a set of textiles with perceptible physical concrete features. These characteristics are the resulting product of particular physical circumstances or some applied techniques.
  - Moiré (attribute): When as an adjective it refers to any fabric with a resemblance or with a moiré effect, that is, a fabric which resembles the rippled effect.
- Cloth area: It refers to any of the parts of a textile, fabric or cloth.
  - Pattern unit: The unit, which is composed of one or more motifs that, repeated, constitute the pattern of a textile.
- Interfunctional elements: All those elements or actions that take part in the weaving process and the relation among them.
  - Binding system: It defines the system in accordance with which ends, and picks are bound.
- Fabric process and production: All those previous processes, with the thread already arranged, that precede the production of the fabric and the actions that imply types of textile production.
  - Shed: n. From the Old-Middle English “schad”. The path across the warp in the loom, that is, the opening made between the threads of the warp by the motion of the heddles for the shuttle to pass through.
- Fabric pre-production: It refers to all those processes that involve the weaving which go from the initial preparation of the thread until the moment before making the textile.
  - Ply: v. To twist together two or more single threads or ends to make a thicker yarn or thread.
- Geographic featured textiles: It describes the way a fabric or textile has been created, the weaving process, the fibres and the tools used, that, in sum, could be known by the denomination of their origin.
  - Pekin: n. A borrowing from the French “Pékin”. From previous forms as “pekin” or “pequin”. It refers to a warp-striped fabric or textile made employing different binding systems and using various fibres including silk, rayon, worsted, cotton or combinations of these fibres. Original from China.

These new guide terms were created based on the Washington Textile Museum [18]. It should be noted that not all of its structure was taken into account as it is a thesaurus created specifically for their collection, while in the SILKNOW thesaurus we had to take into account many data sets coming from several museums, hence not homogenized. As SILKNOW data comes from museums that have silk in their collection not all of them have the same nature (ethnographic museums, fine arts museums, national museums, private collections, textile museums, etc.).

### Multilingualism

The decision to produce the multilingual thesaurus [19] was based on the construction of a symmetrical thesaurus, meaning that every Prefered Term had to have an equivalence in every language (English, Spanish, French and Italian). As stated by [20, 21], the Multilingual Subject is a place located in language, but it is an embodied, socially and culturally inflected place, a place filled with memories of other languages, fantasies of other identities [22] and communicative joys, of a symbolic gamble and subjective power [23]. In this sense, we wanted to avoid the so-called “multilingual writing” which in fact, tends to the drafting of texts in one language, almost always in English, and their subsequent translation into the others. While a true multilingual thesaurus offers complete conceptual and terminological inventories for each one of the languages involved [19]. In a situation of the superiority of a single language, the terminological basis tends to present the concepts and their interrelations as given in that language. This was one of the main issues we wanted to avoid, by introducing all the possible variables from an inclusive and multicultural diversity associated with local cultural heritage and the language policies applied in those regions focused on cultural diversity [24].

In order to develop the SILKNOW thesaurus, a common methodology was established that implied firstly to translate preferred terms. There was an extensive literature review, with 140 references studied in Spanish, 70 for English, French 142 and 36 in Italian. As stated before, it was necessary to present a fully developed structure in every language, so the user could get the same amount of semantic information in the language most appropriate for her or him. In this sense, the literature review allowed not only to translate terms but to add terms in every language, that is, whenever a researcher founded an important concept in his or her language it was immediately added and searched in the rest of languages. By doing it we started from language differences, taking into account their relationship with identity, history and education. Therefore, we based our methodology not only in translating each to term to its equivalent but also establishing, from the sociolinguistic point of view, aspects that have to do with linguistic evolution and the relation among the four languages and the intergenerational transmission of heritage languages [25]. An example is the word “samite”, a historical term from the Middle English “samit” “samet” “samyt” “samyte” among others, which refers to a rich silk fabric, worn in the Middle Ages. The word samite derives from the Latin word examitum, samitum, and from the Greek word hexamitos, six threads. This word has evolved in samitz, çamit, samito, xamet, xamete or jamete, in English is samite, in Spanish is Jamete, in French is Samit and in Italian is Sciamito.

This has been possible by linking disciplines as separate as dialectology and historical linguistics [26] with the art history research (specially focused on silk heritage conservation) and with the classification and cataloguing records of museum institutions and the basis of existing thesauri, such as those already described. In this sense, not all terms had equivalences, whenever it happened the Source Language, was used as a loan in the Target Language in the rest of languages. For example, “anafaya”, as it is a word that comes from Arab and specifies a cloth from Spain, made of cotton or silk. Another example is “liage à repris” a French term that describes the effect this produced by holding the drawing using the binding warp, it is a background warp that ties a decorative weft following a proportion. This term is worth mentioning as we took into account a local term that is only used in the traditional Valencian industry, “restañado”, which was added as a synonym. In this way, a user can look for “restañado” and will find together with the preferred term that is recommended in the literature. Some terms coming from the source language, provided some extra difficulties to establish the equivalence at the meaning was either regional such as the case of espolin, which neither in French nor in English could be translated, so it was left as “espolin”, while in Italian was translated as “spolinato” because the conexions with the Crown of Aragon.

On the other hand, polysemy was also treated in all languages, this means that whenever a word was polysemic its several concepts were added in the thesaurus with qualifiers in order to distinguish them. Then, each researcher added these terms and established the equivalences in the other three languages as shown in Table 3.

**Table 3:** Polysemic words

English	Spanish	French	Italian
Yarn (From the Old English “gearn”. A term used to designate thread prepared for weaving or knitting)	Hilo (hilo)	Fil (fil)	Filo (filo)
Thread (a component of a silk yarn, it is the product of winding together without twist a number of filaments or fibres.)	Hilo (hebra)	Fil (fibre)	Filo (filone)

In this sense, a conceptual thesaurus like SILKNOW, enhances multilingualism as the automatic search of records with these kinds of controlled vocabularies based on descriptors allows not only a proper document indexing and retrieval but also to map texts as the basis for a semantic web [27].

With the advance of information technology, heritage organizations are now managing information in multiple languages that comes from multiple sources and several geographical regions. In these scenarios, cross-lingual information retrieval and cross-lingual text categorization are important tasks for multilingual knowledge management [28]. The SILKNOW thesaurus arises as an important step to preserve, conserve and disseminate silk heritage, with the hope that in the future more languages can be added.

## Relationship with wikidata

To investigate relationships of the SILKNOW thesaurus and existing ontologies, we considered the Wikidata ontology. We started by matching the concepts on the basis of labels: a Wikidata concept is taken to be a candidate match for a SILKNOW concept if they have at least one common label in at least one language. Domain experts then examined these candidate matches manually and evaluated each of them as being either an exact match, a partial match, or a mismatch. On the basis of this evaluation, we may divide the 666 concepts of the SILKNOW thesaurus into several groups shown in Table 4. From each group, 15 randomly chosen examples are also shown. Where the English labels of the concepts are ambiguous, the Spanish label is included in brackets.

**Table 4:** 666 concepts of the SILKNOW thesaurus divided into several groups

Number of SILKNOW concepts	Percentage (%)	Description
290	44.4	SILKNOW concepts with at least one exact match in Wikidata (of these, 100 SILKNOW concepts even had two or more exact matches) Examples: animal fibre, batik, binding warp, cancanias, chinoiserie style, draw loom, denier, edge, filature, flax, foulard, modernism, romanticism, shot, spun silk
39	6.0	SILKNOW concepts with at least one partial match in Wikidata, but no exact matches (the matches were typically evaluated as partial because the Wikidata concept was not quite as specific as the one from SILKNOW) Examples: artichoke, aurora pink, crepe [Sp. *crespón*], crimson, end [Sp. *cabo*], floss, Latin cross, lace [Sp. *encaje*], lace [Sp. *puntas*], liturgical paraments, plush [Sp. *afelpar*], plush [Sp. *felpa*], ply, punched card, warp
75	11.5	SILKNOW concepts where all the candidate matches were evaluated by the domain experts as mismatches (these were mostly concepts where a polysemous word appears as the label, but none of the Wikidata concepts with this label refers to the same sense of that word as the SILKNOW concept does) Examples: barred, bave, cannele, comber unit, crossing, crepe [Sp. *hilo crespón*], faille française, glazed, nuance, printed, reverse, sendal, segrí, silk skein, tissue
249	38.1	SILKNOW concepts for which no candidate matches were generated at all, because there was no Wikidata concept with an identical label. For concepts in this group, we do not know if an equivalent concept exists in Wikidata or not; it is possible that an equivalent concept exists but under a completely different label Examples: atractiva, broderie velvet, button drawloom, continuous yarn, crepe de Lyon, gilt membrane strip, gregoire velvet, inverted mirrored disposition, lampas taille-douce, louisine, queen satin, spiral thread, trama interrumpida, vegetal motif, warping person

We can conclude that at least 44% of the concepts from the SILKNOW thesaurus are also present in the Wikidata ontology; it is possible that the true percentage is higher because some concepts in Wikidata might not have any matching labels with their counterparts in the SILKNOW thesaurus. However, as can be seen from the examples in Table 4, the concepts from the last group (for which there is no Wikidata concept with the same label in any language) tend to be more specific, with longer, multi-word labels, and they are thus less likely to appear in Wikidata.

## Evaluation

### Coverage studies

Thesaurus coverage was calculated for different versions of SILKNOW thesaurus. Data in this article is based upon SILKNOW thesaurus 1.8 version. To better understand how the developed thesaurus covers the resources that we have included in the project, we have performed an analysis of the final version of the thesaurus that will be available on the web, regarding coverage in different languages and number of unused terms.

#### Usage of the thesaurus concepts in the museums

**Fig. 1:**
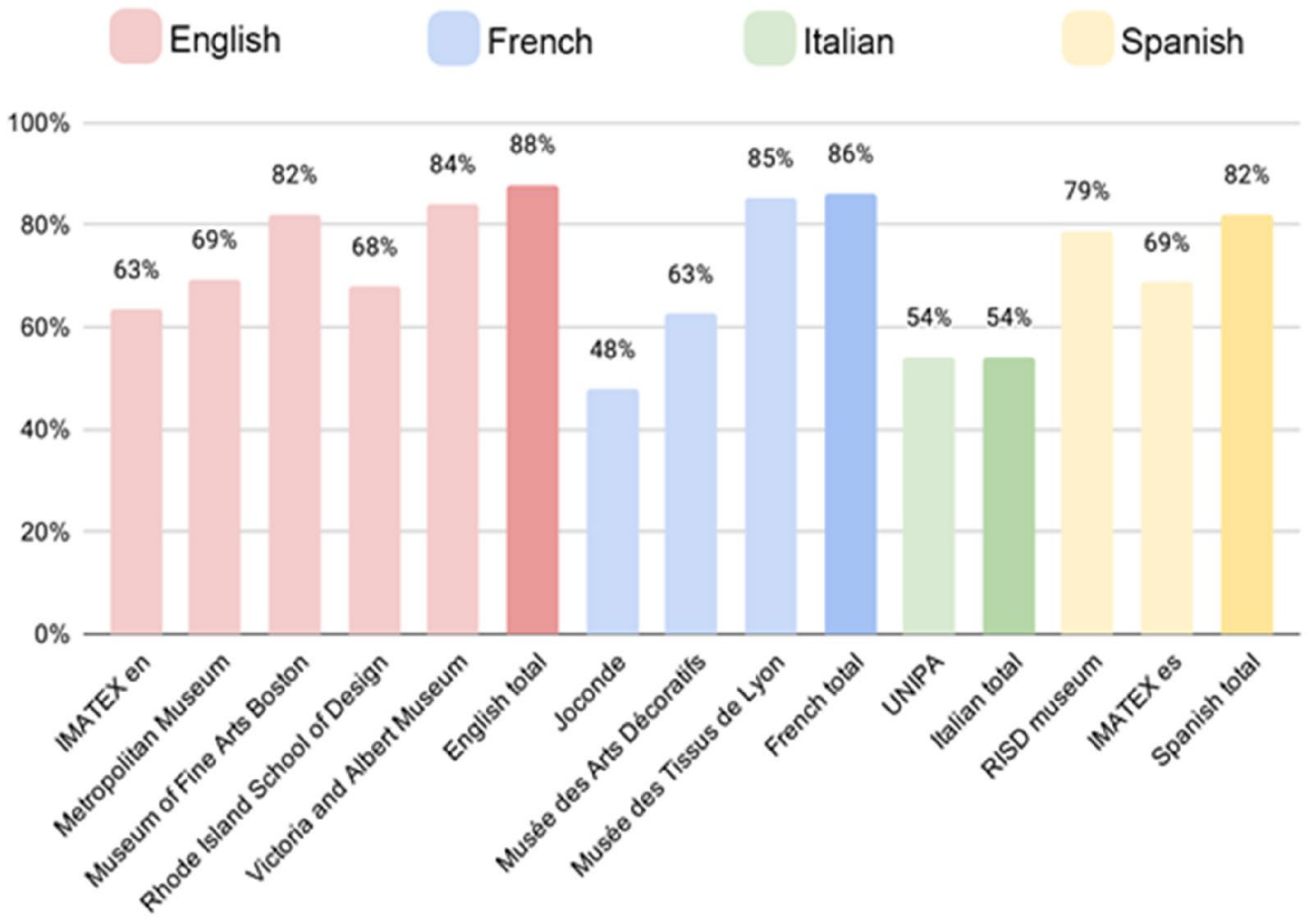
Percentage of SILKNOW thesaurus concepts that occur in individual museums in the corresponding language

**Fig. 2:**
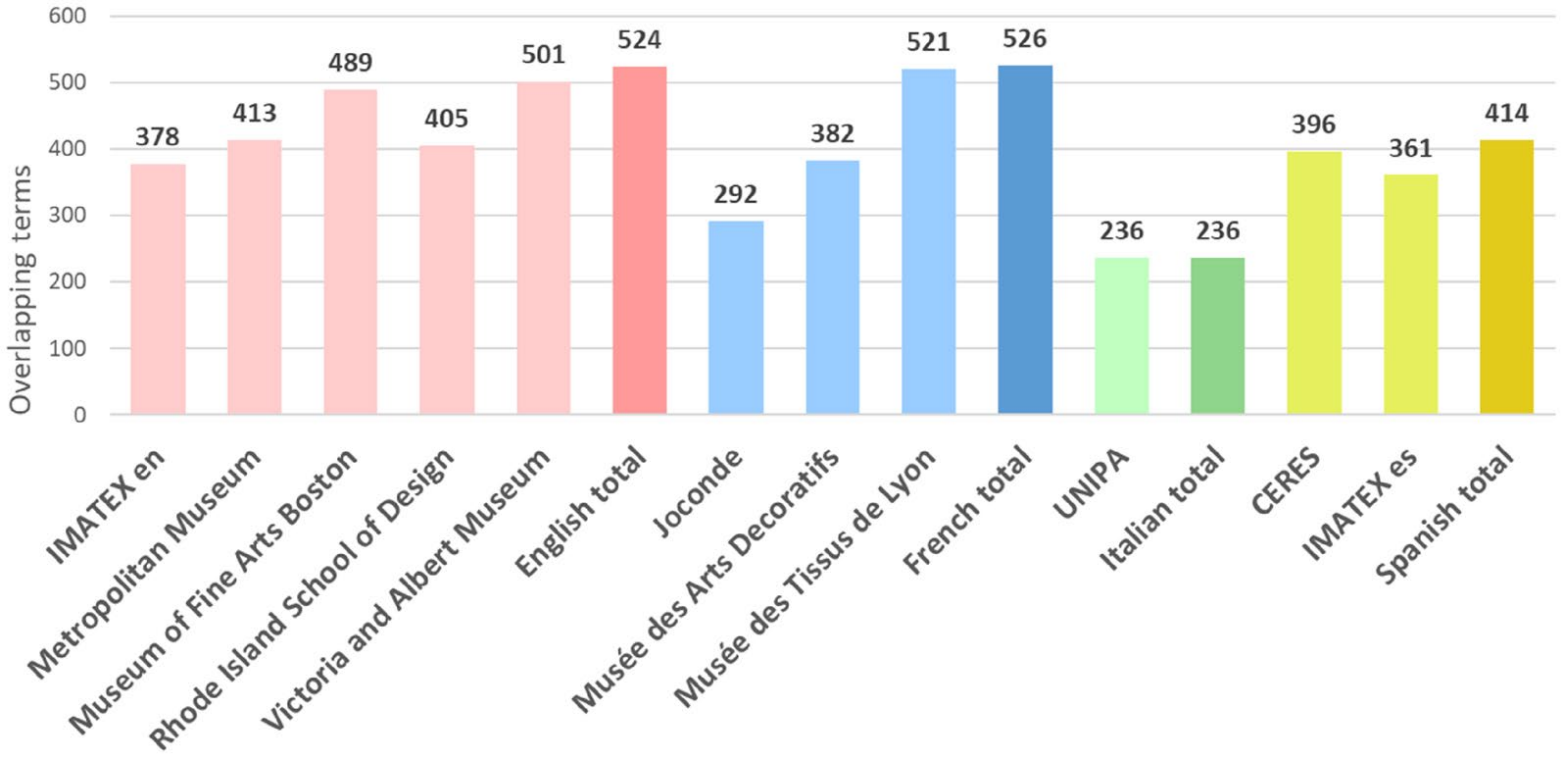
The number of overlapping terms of SILKNOW thesaurus and the individual museums

The SILKNOW thesaurus was validated on textual data of online resources on the full thesaurus in all four languages it covers. The frequency of the individual thesaurus concepts that are present in specific online resources was calculated. English, French, Italian and Spanish translations of the thesaurus were each compared to resources in the corresponding language. The program for the calculation of coverage was written in Python. Preprocessing was carried out using the Natural Language Toolkit library which contains the Snowball Stemmer. It was used to convert all the terms and their synonyms from the thesaurus, as well as all the words from online resources, to their stem. This enabled us to compare two words and determine whether they have the same stem. The output of the coverage calculation is a table, where each row contains a term, its synonyms and the number of times the term was used in one of the resources. From this, the percentage of thesaurus concepts that can be found in the specific resources was calculated. The percentages are shown in Fig. 1 and the exact numbers are given in Fig. 2.

The English part of the SILKNOW thesaurus contained at the moment of this experiment, 596 concepts. Out of these, 72 do not occur in any of the English museums that we have considered. These include: cannetillé, aceytuni, alberoni, alcarchofado, fibroin, pill, lammela, monture, etc. The French part of the SILKNOW thesaurus contained 611 concepts. Out of these, 85 do not occur in any of the French museums that we have considered. These include: artichaut, pélican, etoile, mante, ourdisseur, tercianela, nobleza, filoselle etc. The Italian part of the SILKNOW thesaurus contained 436 concepts. Out of these, 200 do not occur in any of the Italian museums that we have considered. These include: mantello, collarino, arazzo, restagno, piombo, tulle, sericina, etc. Finally, the Spanish part of the SILKNOW thesaurus contained 629 concepts. Out of these, 113 do not occur in any of the Spanish museums that we have considered. These include: zarza, frappé, tusor, devanador, rasete, sericina, lisaje, estofar, etc.

**Fig. 3:**
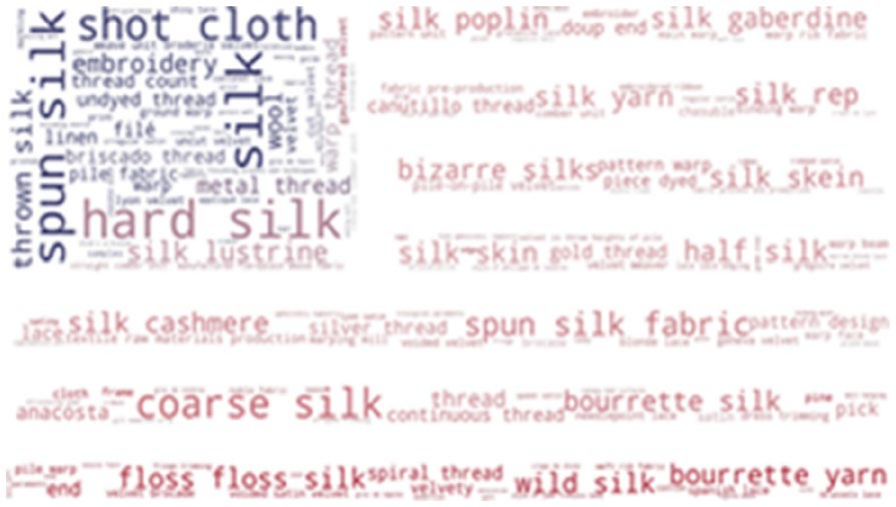
Usage of SILKNOW thesaurus concepts in Metropolitan Museum

**Fig. 4:**
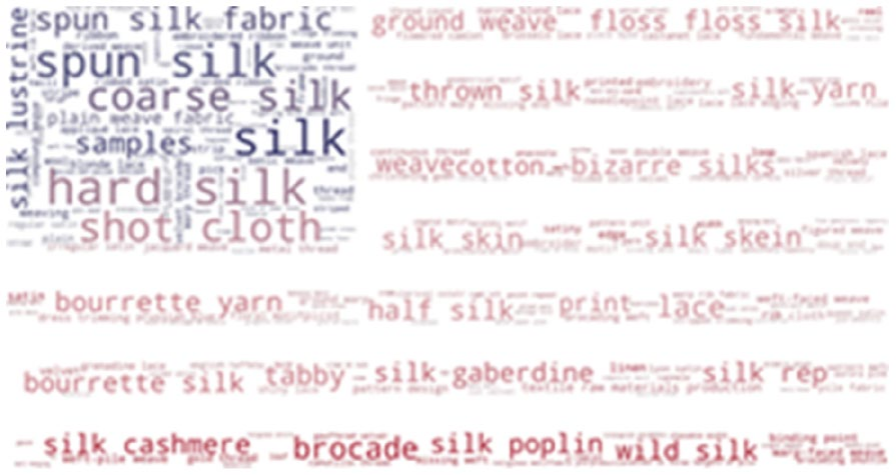
Usage of SILKNOW thesaurus concepts in Museum of Fine Arts Boston

To investigate which terms from the thesaurus are the most frequently used in our resources, we provide word-cloud visualization. As shown in Fig. 3, some of the most frequently found thesaurus concepts in Metropolitan museums are: hard silk, spun silk, shot cloth, coarse silk, half silk and floss silk. The list of the most frequent concepts in the Museum of Fine Arts, Boston is very similar: hard silk, spun silk, shot cloth, coarse silk, tabby and bourette yarn (Fig. 4).

**Fig. 5:**
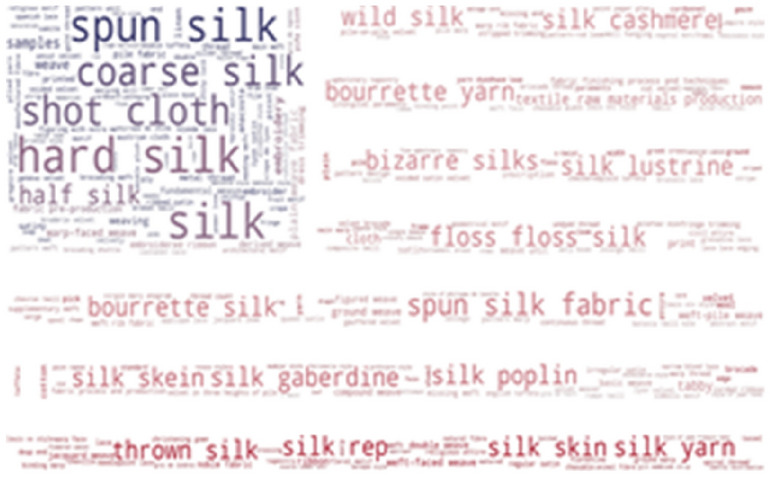
Usage of SILKNOW thesaurus concepts in Rhode Island School of Design

**Fig. 6:**
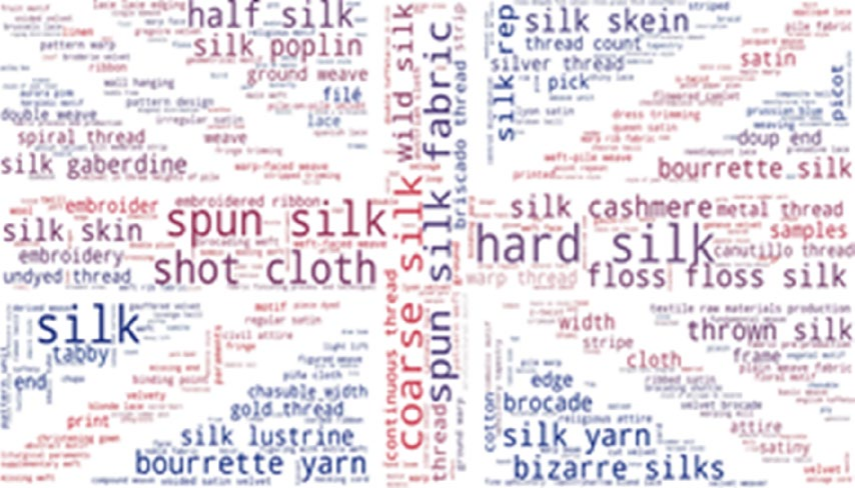
Usage of SILKNOW thesaurus concepts in Victoria and Albert Museum

Some of the most frequently found thesaurus concepts in the Rhode Island School of Design are hard: silk, spun silk, shot cloth, coarse silk and bourette yarn (Fig. 5), while some of the most frequently found thesaurus concepts in the Victoria and Albert Museum are: hard silk, spun silk, shot cloth, coarse silk and half silk (Fig. 6).

**Fig. 7:**
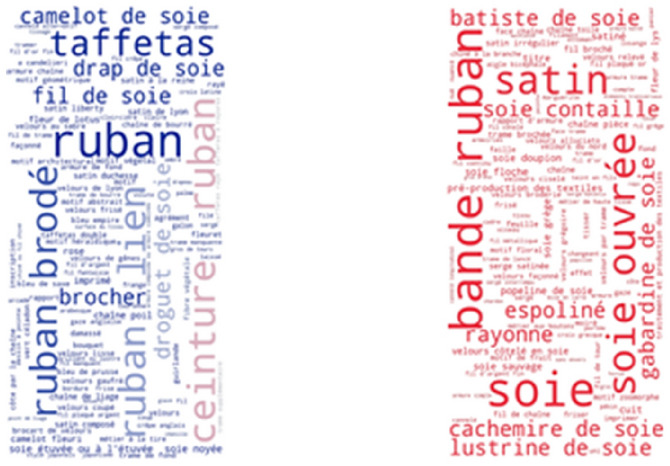
Usage of SILKNOW thesaurus concepts in Joconde

**Fig. 8:**
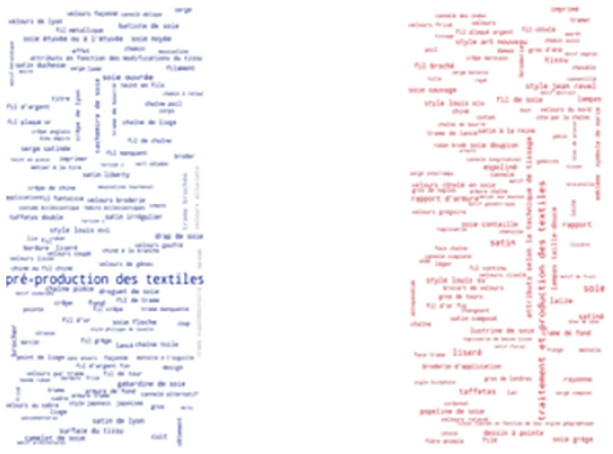
Usage of SILKNOW thesaurus concepts in Musée des Arts Décoratifs

Interestingly, the most frequent thesaurus concepts that occur in French resources are quite different from the most frequent English concepts. As shown in Fig. 7, some of the most frequently found thesaurus concepts in the Joconde database are: ruban, bande, soie, ruban brodé, ceinture and rayonne. These French concepts translate into English as: ribbon, loop, silk, embroidered ribbon, girdle and rayon. As shown in Fig. 8, some of the most frequently found thesaurus concepts in the Musée des Arts Décoratifs are: pré-production des textiles, laize, soie, soie ouvrée, tissu and brocher. These French concepts translate into English as: fabric pre-production, width, silk, thrown silk, cloth and brocade. Notice that the range of frequencies of individual concepts is low, resulting in less variable font sizes.

**Fig. 9:**
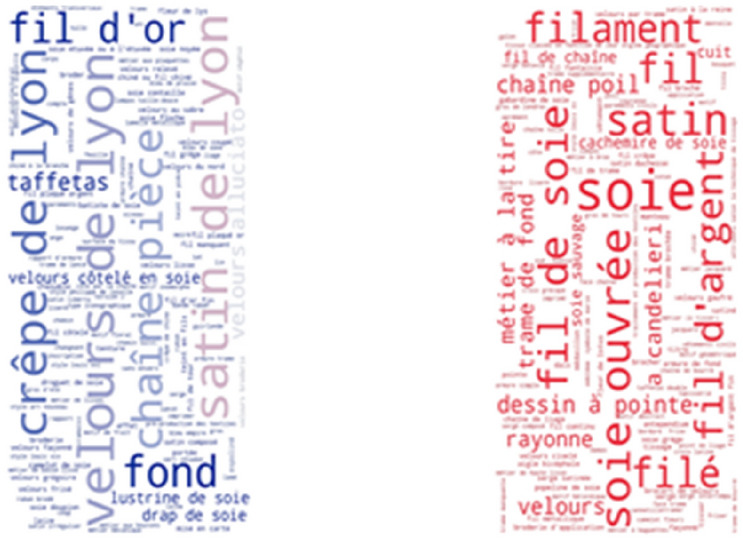
Usage of SILKNOW thesaurus concepts in Musée des Tissus de Lyon

**Fig. 10:**
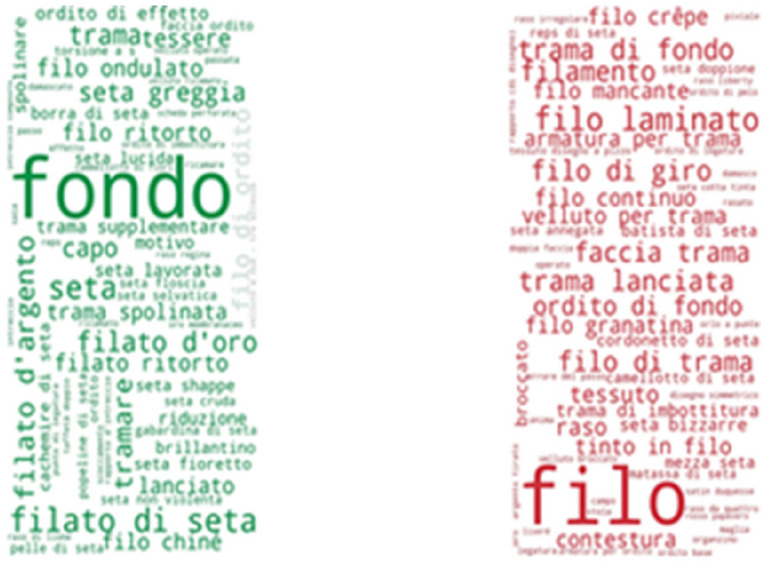
Usage of SILKNOW thesaurus concepts in UNIPA

Some of the most frequently found thesaurus concepts in the Musée des Tissus de Lyon are: velours de Lyon, satin de Lyon, crêpe de Lyon, soie, chaîne pièce, fond (Fig. 9). In English these are: Lyon velvet, Lyon satin, Lyon crape, silk, main warp and ground. Some of the most frequently found thesaurus concepts in UNIPA are: fondo, filo, filato di seta, trama di fondo, trama lanciata and filo di giro (Fig. 10). These Italian concepts translate into English as: ground, yarn, silk yarn, main weft, pattern weft, doup end.

**Fig. 11:**
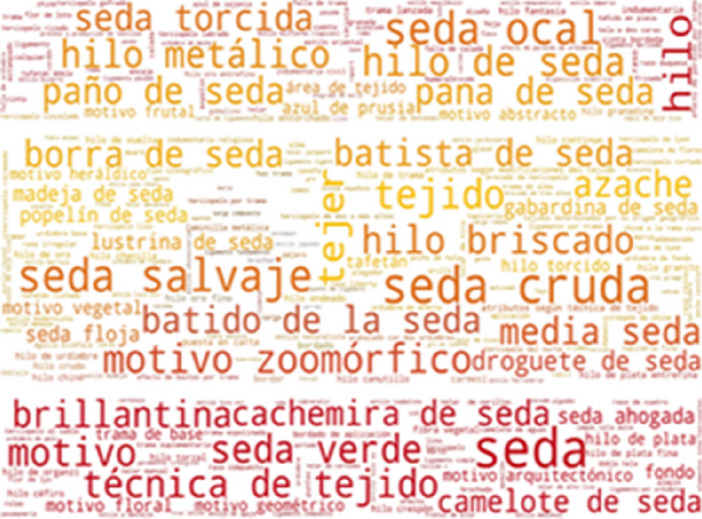
Usage of SILKNOW thesaurus concepts in CERES

**Fig. 12:**
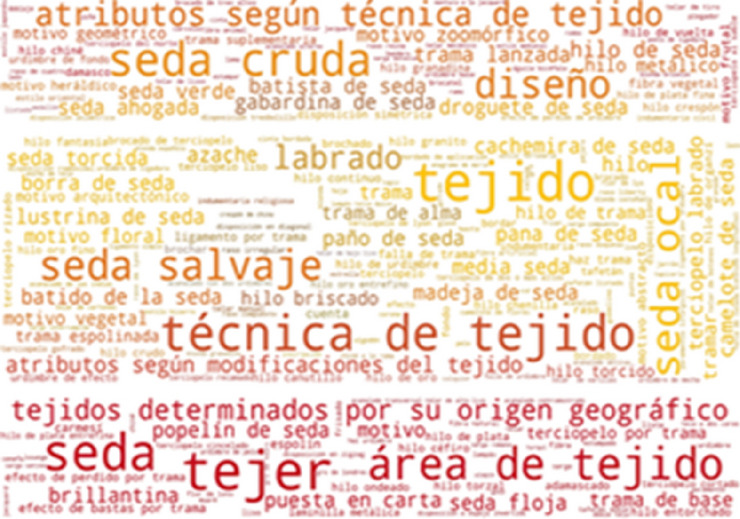
Usage of SILKNOW thesaurus concepts in IMATEX

Some of the most frequently found thesaurus concepts in CERES are: seda, seda cruda, seda salvaje, hilo de seda, tejido, tejer and motivo zoomórfico (Fig. 11). These Spanish concepts translate into English as: silk, hard silk, wild silk, silk yarn, cloth, weave and zoomorphic or animal motif. As can be seen in Fig. 12, some of the most frequently found thesaurus concepts in IMATEX are: tejer, tejido, seda cruda, seda salvaje, labrado and diseño. These Spanish concepts translate into English as: weave, cloth, hard silk, wild silk, figured weave and pattern design.

#### Word and phrases in the museums not covered in SILKNOW thesaurus

For each online resource, a feature vector representing all its phrases was computed. The result was a set of n-grams with a maximum size of three words and a corresponding number of occurrences. From them, a subset was generated where all the concepts that can be found in the thesaurus were removed from the feature vector. This subset is referred to as online resource phrases without thesaurus overlap.

**Fig. 13:**
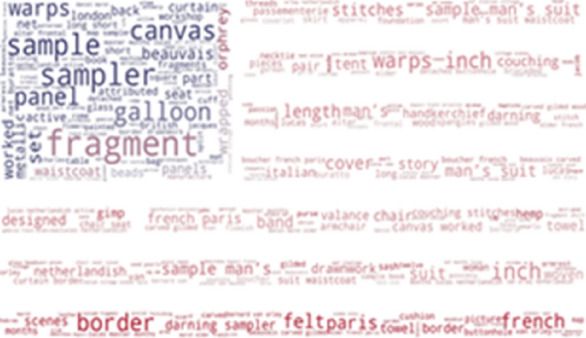
Metropolitan Museum phrases not covered in SILKNOW thesaurus

**Fig. 14:**
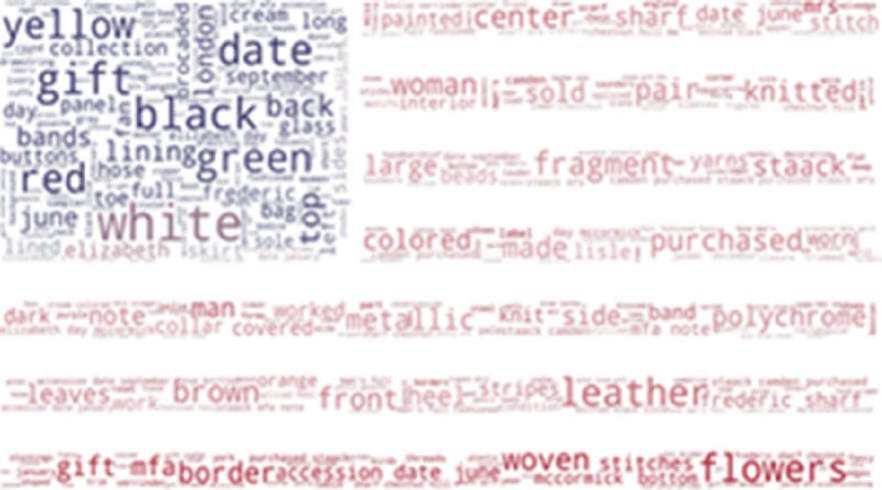
Museum of Fine Arts Boston phrases not covered in SILKNOW thesaurus

Figures 13 and 14 show the most frequently found phrases not included in the SILKNOW thesaurus on the Metropolitan Museum and the Boston Museum of Fine Arts such as fragment, sample, canvas or panel. From this, we can presume that the SILKNOW thesaurus covers most domain specific terminology as these are common everyday words or easily understandable terms.

**Fig. 15:**
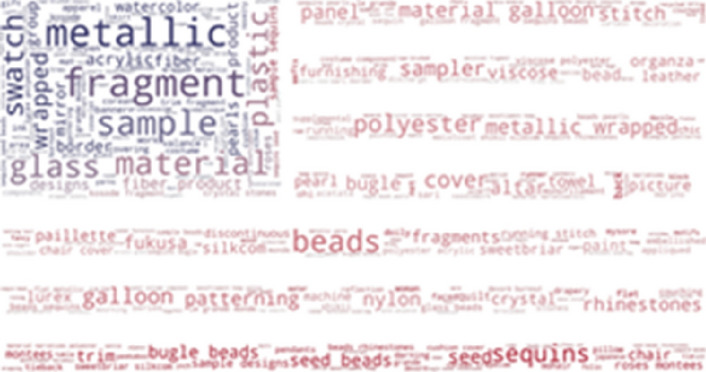
Rhode Island School of Design phrases not covered in SILKNOW thesaurus

**Fig. 16:**
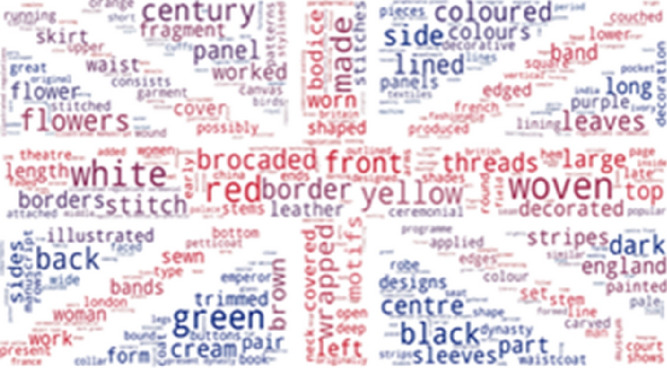
Victoria and Albert Museum phrases not covered in SILKNOW thesaurus

Regarding the Rhode Island School of Design (Fig. 15) and the Victoria and Albert Museum (Fig. 16), we find the same results as with the previous museums, including common colours such as, yellow, red, white or green. We can assume that the SILKNOW thesaurus covers most domain specific terminology in English language.

**Fig. 17:**
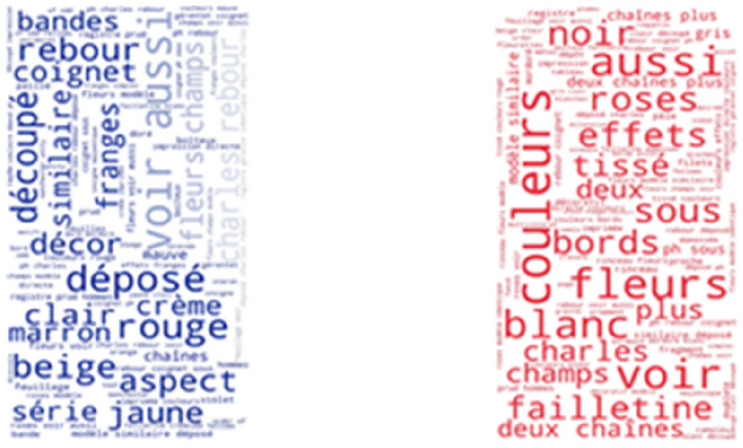
Joconde phrases not covered in SILKNOW thesaurus

**Fig. 18:**
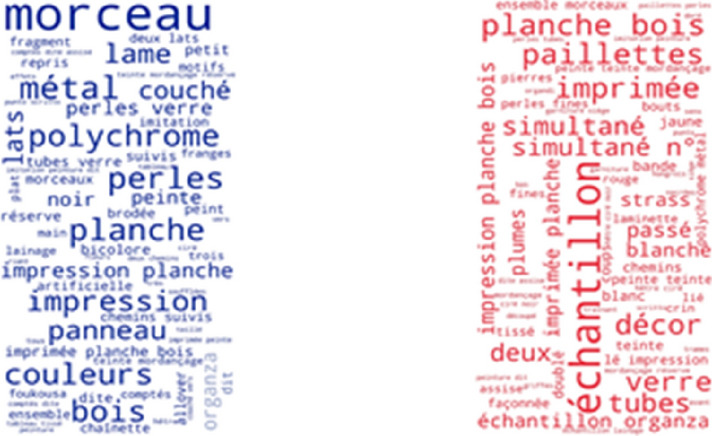
Musée des Arts Décoratifs phrases not covered in SILKNOW thesaurus

**Fig. 19:**
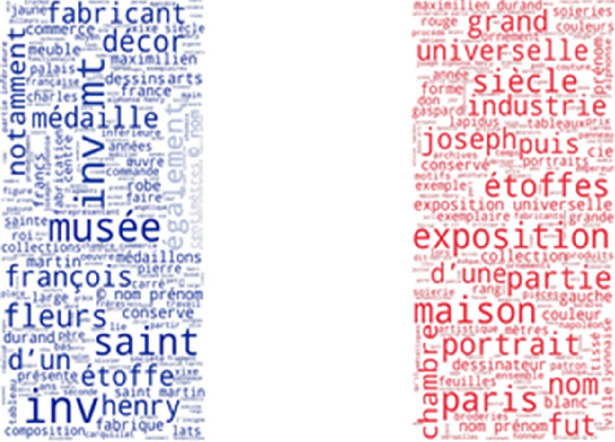
Musée des Tissus de Lyon phrases not covered in SILKNOW thesaurus

As it is a multilingual thesaurus, we also wanted to know the most frequently found phrases not included in the SILKNOW thesaurus in other languages. In French, we looked at the Joconde database (Fig. 17) and the Musée des Arts Décoratifs (Fig. 18) and Musée des Tissus de Lyon (Fig. 19). These include: colors, flowers, samples, museum and manufacturers among others. As it happens with English, these data us mostly represented by common everyday words or easily understandable terms. From this we can presume that the SILKNOW thesaurus covers most domain specific terminology used in French museums and databases.

**Fig. 20:**
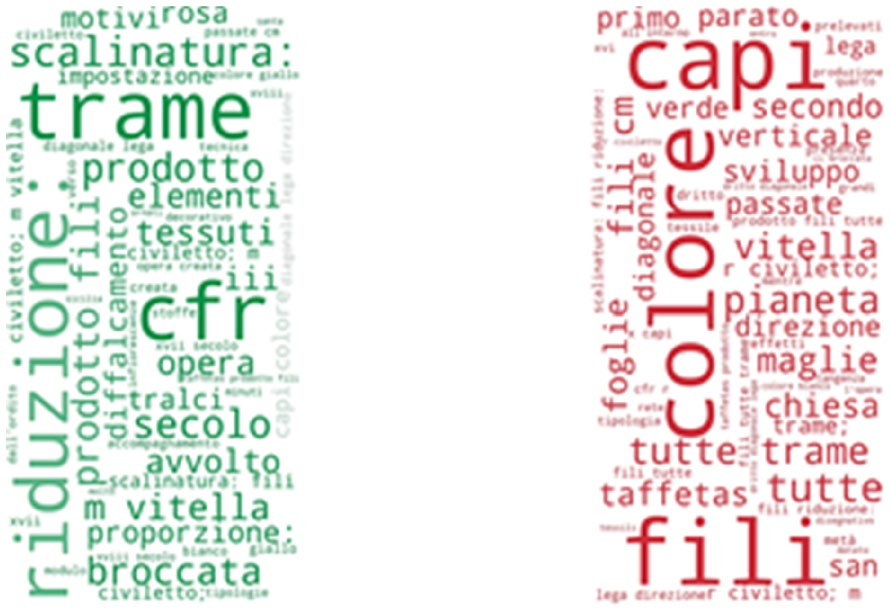
UNIPA phrases not covered in SILKNOW thesaurus

As per Italian, the most frequently found UNIPA (Fig. 20) phrases not included in the SILKNOW thesaurus are: trame, capi, colore, fili, riduzione, secolo and prodotto. These Italian phrases translate into English as: weave, leaders, colour, thread, reduction, century and product. Data is mostly represented by common everyday words or easily understandable terms. From this, we can presume that the SILKNOW thesaurus covers most domain specific terminology used in the UNIPA dataset. Additionally, sacred motifs are also evident from the high frequency of the phrases: chiesa, san, verde, tralci and foglie, which translate as: church, saint, green, vines or branches and leaves.

**Fig. 21:**
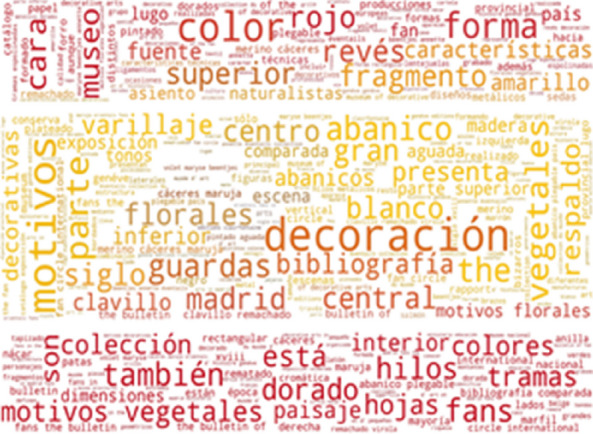
CERES phrases not covered in SILKNOW thesaurus

**Fig. 22:**
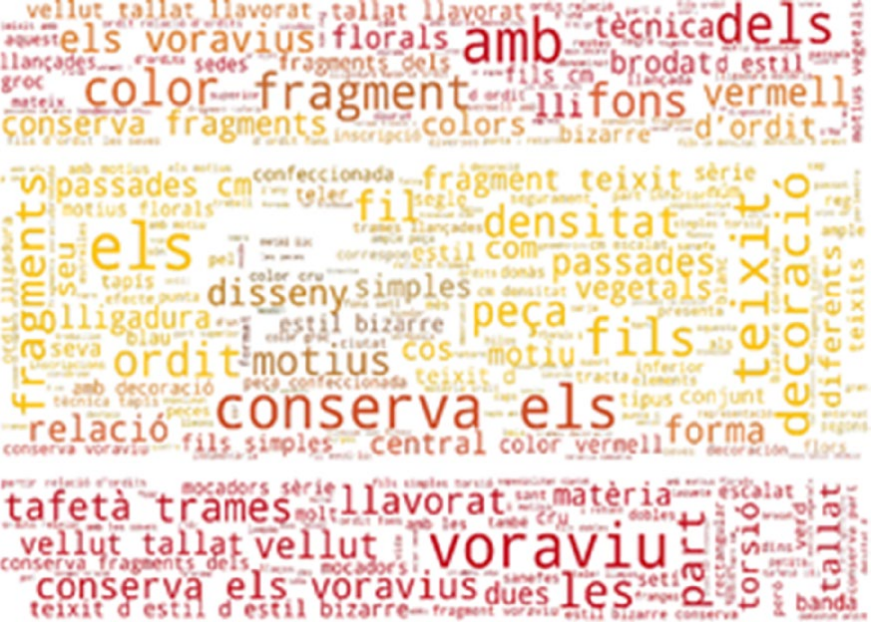
IMATEX phrases not covered in SILKNOW thesaurus

Finally, as can be seen in Figs. 21 and 22, some of the most frequently found CERES and IMATEX phrases not included in the SILKNOW thesaurus are related to colors, bibliography, shapes or state of conservation, hence, we can concur that the SILKNOW thesaurus covers most domain specific terminology used in the four languages it was built, becoming and important asset for conservation purposes.

#### SILKNOW thesaurus concepts not occurring in data

For each of the four languages there are some SILKNOW thesaurus concepts that were not found in data from online resources. Table 5 provides percentage of such concepts in SILKNOW thesaurus for each language. These concepts might be missing because they are part of very domain specific terminology related to silk. Given that some of the concepts are tied only to specific time periods or places, it is unlikely for them to be used fully in every online resource, but they might still be included in the resources added in the future. The following are two examples of SILKNOW thesaurus concepts which were not found in online resources. The first example of the concept “damaras”, which is defined as: “A thin and lightweight taffeta, which has patterns of flowers. It comes from India.”, shows how a concept can be tied to a specific place. The second example of the concept “aceytuni”, which is defined as: “A medieval name for satin. Rich silk fabric that began to be manufactured in Chinese town of Zayton…”, shows how a given concept can also be tied to a specific time period. In this regard, we can say that there are some concepts that are related to a specific area such as “restañado” used only in the Valencian region (not even in the entire Spanish national region), however, thanks to synonyms this concept can be found. In fact, this concept uses “liage repris” as Preferred Term in every language.

**Table 5:** The absolute number and percentage of SILKNOW thesaurus concepts for each language separately, that do not occur in the data from the considered museums

SILKNOW thesaurus concepts not occurring in the museums			
Language	No	[%]	Example concepts
English	72	12.08	Tetramorph, zoomorphic, ternum, poult, tercianela and sericulture
French	85	13.91	Denim, chrisme, etoile, pourpoint, mante and rhadamés
Italian	200	45.87	Casula, terno, collarino, tovaglia, arcata and batavia
Spanish	113	17.97	Zarza, fular, muaré, rádium, armura and devanar

We can see the percentage of unused concepts is the lowest for English, followed by French, Spanish and Italian. This might be simply because there are four English online resources included in this study, compared to three French, two Spanish and one Italian. One would expect that including more resources will decrease the number of unused concepts.

### Domain expert’s validation

The SILKNOW thesaurus was validated following a structured methodology that began with an extensive literature review, that is, a scope note was only approved after reviewing at least three authoritative sources. In this sense, the thesaurus has more than 278 references. The literature reviewed has been divided into:

- General dictionaries: These include each reference source of each language (RAE in Spanish; Oxford in English; Dictionnaire de l’Académie française in French; Trenccani in Italian). These dictionaries were very useful to select PT.
- Specialized dictionaries: These dictionaries cover the domain of silk heritage and were used to better define and select specific terms related to historical silk.
- Specialized references: Specialized books served both because of their glossaries of specific terms, and because they improve knowledge of historical fabrics.
- Historical sources: They helped the researchers to define historical terms, their evolution over time and their current use.
- Thesauri: Other thesauri were barely used as they are quite generic.
- Journal papers: Academic literature was reviewed due to its high degree of specialization.
- Thesis: Ph.D. thesis were also consulted.

**Fig. 23:**
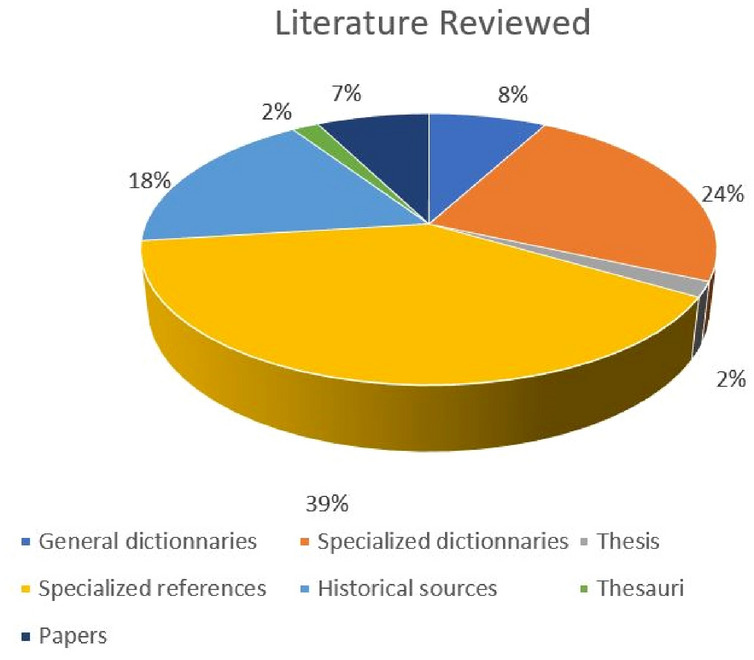
Specialized literature used in the thesaurus

Figure 23 shows the total of the literature review.

The second step was to review the SILKNOW ontology that as per May 2021 it has more than 40,000 instances. In order to do so, statistics were provided by EURECOM. In this sense, it is important to remark how a multidisciplinary team can make the difference. This information on many occasions provided terms that appeared many times in data, but are not really specific within textile proper terminology, such as *patterned fabric*, a generic term that refers to any fabric that has decoration and figurative elements. On other occasions, reviewing literature led to adding terms that are not frequent in data collections but are quite specific such as *tiraz*.

Next steps involved discussion with other domain experts including weavers, art historians, historians and philologues. One of the most important steps was to discuss terms with the translators of the SILKNOW thesaurus, as mentioned, they were part of the SILKNOW thesaurus. Here it was necessary to confront not only terms in the four languages, but also to confront the existing literature in those languages.

Finally, the thesaurus was distributed in workshops held for museums with textile collections, which, on the one hand, showed how the main problem of textile conservation is the standardization of vocabulary and the lack of a specialized thesaurus. On the other hand, these museums and their textile specialists have constantly reviewed the terms, giving feedback to researchers, who are constantly improving the silk thesaurus.

This methodology guaranteed that the SILKNOW thesaurus was improved on many occasions, hence it is scientifically accurate and is the only existing open-access, interoperable, silk heritage thesaurus.

## Conclusions

GLAMs and Cultural Heritage institutions aim to conserve and disseminate their collections; to do so, prior knowledge is extremely important; the registration of a cultural asset in an inventory or its inclusion in a catalogue assumes its recognition as an element that is worth conserving and protecting for future generations. Controlled vocabularies stand out as essentials to provide access to museum collections not only to inside users (curatorial staff, conservators, education department), but also to external users who wish to know more about a subject without knowing the specific term of its search [5]. This thesaurus is not only intended as a scientific prop, but also it plays a fundamental role for the protection and conservation of the vast and fragile silk heritage, in its materiality (objects) and its intangible aspects (weaving techniques).

The SILKNOW thesaurus arises as a very much needed tool to protect this heritage, not only by properly naming it, but also to allow connections among collections in time and space. It covers regional terminology but also terminology that is commonly used in silk industries but not in silk museums, as well as includes styles and periods that arise as fundamental when cataloguing an object. In this sense, not only cultural heritage professionals will be benefited from the thesaurus but also creative industries, allowing the interdisciplinarity that characterizes the SILKNOW project.

To build this thesaurus, authors had to work with an interdisciplinary point of view, contributors were philologists, art historians, historians, museums conservators, weavers and silk merchants. The result of this team is a thesaurus that covers not only the most frequent concepts used in museums but also those that are used in academic papers and in the current and traditional silk industries. This means that we are conserving tangible and intangible silk cultural heritage memory. In this regard, one of our recommendations for future thesauri is to be as multidisciplinary as possible.

In this sense, applying computational methods to build these types of thesauri are extremely useful, as they help developers to discover the terms that are most often used in museums. It is fundamental to know tho they catalog as developers could include terms that were not quite specific but often used, such as *patterned*, which designates any decorated textile with a repeated pattern.

On the other hand, future work goes through the continuous revision of the current included concepts with the recent literature. In this regard, working during the COVID-19 pandemic was a challenge. Open access information is an urgent need as demonstrated by [29]. Indeed, if we do not have access to dictionaries, glossaries, doctoral theses, journal papers, etc., it would be more difficult to develop new knowledge.

Additionally, we would like to incorporate more concepts coming from other languages such as German or Dutch (for their tradition in weaving and selling silk). This would be a huge challenge as it would require translating the current concepts in those languages, but more difficult would be whenever a term is polysemic in those languages and not in the existing ones. We would have to add those new concepts into the existing thesaurus and create the relations between them, not an impossible task but a complicated one. Finally, it would be recommended to improve SKOSMOS with a more visual design that could include images so users not familiarized with silk can understand concepts.

In this regard, cultural heritage should not be understood only from an economic point of view, but as a means to transmit the legacy of our past. In fact, most current studies highlight the importance of making these values accessible to all citizens. SILKNOW offers an open access tool, which aims to have a strong impact not only on museum collections and researchers, but also on the general public. That is, not only for research purposes, but also for cultural dissemination. In this regard, the thesaurus also connects the past with the future, through making accessible a forgotten past and turning it into an innovation agent.

## Data Availability

Not applicable.
